# Achieving magnetic control of cellular events by introducing non-native radical pair formation

**DOI:** 10.1093/nsr/nwae145

**Published:** 2024-04-15

**Authors:** Jonathan L Sessler

**Affiliations:** Department of Chemistry, The University of Texas at Austin, USA

Migratory songbirds have a magnetic compass that helps them navigate enormous distances. The biophysical mechanism of this compass is not yet known but is thought to involve photochemical intermediates known as radical pairs (RPs) [1]. Although laboratory studies have established the importance of RP effects in the context of chemical systems [2], it has proved challenging to harness these effects to achieve specific goals in biology. On the other hand, it is important to appreciate that, even though studies of RP effects in biomolecular systems have yet to reveal reproducible magnetic field effects (MFEs), extrapolation of the negative findings to the cellular or whole-organism level is ill-advised due to the complexity of biological environments, the homeostatic buffering seen under physiological conditions and other cooperative phenomena that we do not yet fully understand. In a recent publication in *National Science Review*, Zhang, Gao and Yang outlined a bottom-up strategy to exploit MFEs in living cells by translating knowledge obtained from *in vitro* studies of RP chemistry [3].

Aberrant redox signaling underlies the pathophysiology of many diseases. Moreover, reactive oxygen species (ROS) have evolved as important regulators of those signaling pathways. ROS are oxygen-containing molecules with high reactivity. This class of molecules includes superoxide (O_2_^•−^) and hydroxyl (^•^OH) radicals and non-radical species, such as singlet oxygen (^1^O_2_) and hydrogen peroxide (H_2_O_2_). Modulating ROS levels, by either rebalancing the systemic redox environment or inducing oxidative stress, is an attractive approach to disease treatment. On the other hand, oxygen and the related ROS have multiple spin states from triplet, doublet to singlet; this provides a golden invitation, so to speak, to investigate their transformation through spin chemistry. Magnetic fields can break the normally degenerate three spin sublevels of the triplet via Zeeman splitting, which changes the oscillation rate between singlet and triplet states of the RP. Each spin state leads to the production of different ROS. As a result, MFEs can affect the choice of reaction pathway, as well as the reaction kinetics and products’ yields. Indeed, external magnetic fields provide a way to control the redox balance by modulating the spin states of RP intermediates in redox reactions [4]. However, there is no clear explanation for the underlying mechanisms behind these phenomena. As a result, the application of magnetic fields in disease treatments remains limited. This is mainly due to the far smaller energies for MFEs than thermal energies, and the mutual interference among different biological processes [5].

**Figure 1.**
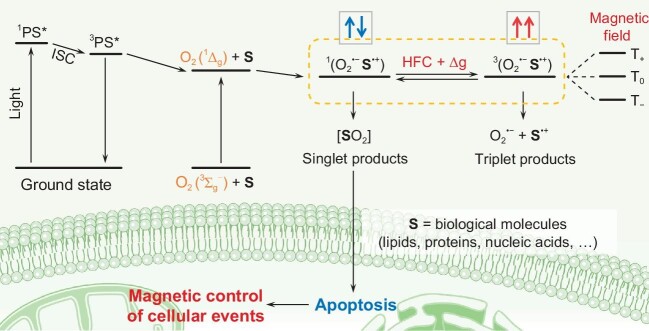
Reaction scheme for magnetic control of biological processes based on exogenous radical pair formation via singlet oxygen as proposed by Zhang *et al*. [3].

Zhang *et al*. [3] proposed a ‘bottom-up’ strategy for exploiting the MFE of chemical reactions in living cells and *in vivo* [Fig. 1]. They started with the reactions of photochemically generated singlet oxygen (^1^O_2_) with electron-rich substrates (iodide, anthracenes) in which the RP (S^•+^ O_2_^•−^) or biradical (^•^SOO^•^) intermediates were found to have an unusual tri-phasic ‘down-up-down’ magnetic dependence in the range of 0–800 mT. Similar patterns were found for biologically relevant lipid peroxidation in organic media, in cell membrane mimics (giant unilamellar vesicles) and in living cells. This work thus shows that magnetic fields can be used to manipulate ^1^O_2_-induced cytotoxicity and cell apoptosis-related protein expression. The results culminated in a proof-of-concept demonstration that applied magnetic fields synergistically to facilitate photodynamic therapy in which ^1^O_2_ is used to kill cancer cells. This work thus highlights the potential of RP mechanisms in the context of magneto-medical drug discovery and development.
